# Beneficial Effects of *Alchemilla vulgaris* in DHEA-Induced Rat Model of Polycystic Ovary Syndrome

**DOI:** 10.1007/s43032-025-01885-9

**Published:** 2025-06-23

**Authors:** Zeynep Ece Utkan Korun, Semil Selcen Gocmez, Selenay Furat, Kubra Kavram Sarihan, Fatma Ceyla Eraldemir, Huseyin Askin Akpulat, Deniz Sahin, Sule Yildiz

**Affiliations:** 1 Department of Obstetrics and Gynecology, Yeditepe University Kozyatagi Hospital, Istanbul, Turkey; 2 Institute of Health Sciences, Department of Stem Cell, Kocaeli University, Kocaeli, Turkey; 3 Faculty of Medicine, Department of Pharmacology, Kocaeli University, Kocaeli, Turkey; 4 Faculty of Medicine, Department of Histology and Embryology, Kocaeli University, Kocaeli, Turkey; 5 Faculty of Dentistry, Department of Basic Medical Sciences, Kocaeli Health and Technology University, Kocaeli, Turkey; 6 Faculty of Medicine, Department of Biochemistry, Kocaeli University, Kocaeli, Turkey; 7 Faculty of Education, Department of Biology, Sivas Cumhuriyet University, Sivas, Turkey; 8 Faculty of Medicine, Department of Physiology, Kocaeli University, Kocaeli, Turkey; 9 Faculty of Medicine, Department of Obstetrics and Gynecology, Koç University, Istanbul, Turkey

**Keywords:** Polycystic ovary syndrome, *Alchemilla vulgaris*, Thoracic aorta, Vascular function, İnflammation

## Abstract

*Alchemilla vulgaris* has traditionally been used to treat gynecologic diseases. The anti-inflammatory and vasorelaxant effects of the extract of *A. vulgaris* (AVE) have been reported in previous studies. This study aimed to investigate the effects of *A. vulgaris* on PCOS, one of the most common metabolic disorders in adult women of reproductive age, accompanied by inflammation and vascular endothelial dysfunction, in an experimental animal model. Three groups of prepubertal female Wistar rats were established: PCOS, Control, and PCOS + AVE groups. Subcutaneous dehydroepiandrosterone (DHEA) was administered for 35 days to the PCOS and PCOS + AVE groups. The rats in the PCOS + AVE group were orally administered AVE (50 mg/100 g bw/day) for 35 days. Systemic blood pressure, vascular endothelial function, ovarian morphology, and endocrine biomarker levels were measured at the end of the experiment. TNF-α levels were also evaluated in the thoracic aorta and ovaries. Hormonal imbalances, ovarian morphological changes, and endothelial dysfunction were observed in the PCOS group, which returned to the controls after AVE treatment. Furthermore, AVE attenuated the aortic and ovarian levels of TNF-α in the PCOS group. Our study suggests that AVE may have beneficial effects in the treatment of PCOS due to its anti-inflammatory and vasculoprotective properties.

**Graphical Abstract:**

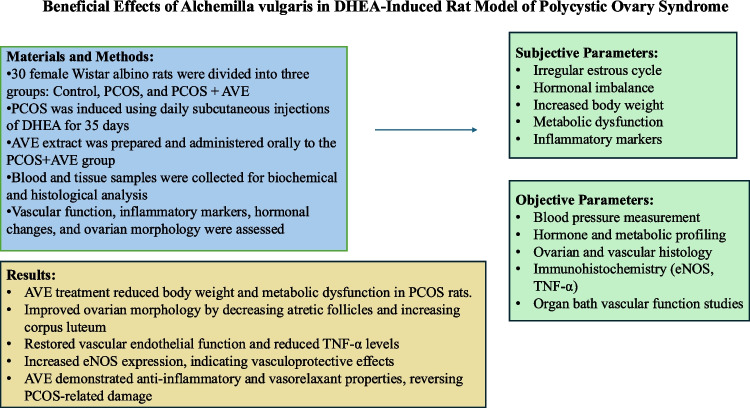

## Introduction

*Alchemilla* is a genus of plants (herbs) in the family Rosaceae. The genus *Alchemilla* L. is reported to comprise 1,000 species with traditional medicinal uses. Numerous studies on the traditional use of *Alchemilla* species indicate various medicinal pharmacological effects, such as antioxidant, antimicrobial, and anti-inflammatory effects, as well as their use in the treatment of endocrine and gynecological diseases [1]. In Turkey, *Alchemilla* species are traditionally used as diuretic and laxatives [2]. However, *Alchemilla vulgaris*, a well-known species of the *Alchemilla* genus, is widely used in folk medicine for the treatment of dysmenorrhea, menorrhagia, and menopausal complaints in various countries [1, 3, 4]. In Palestinian folk medicine, *A. vulgaris* is also used for the treatment of female infertility [5]. Although *A. vulgaris* is widely used as a folk remedy for female hormonal imbalances and gynecological diseases in many countries, to our knowledge, no study has yet reported on its use for the treatment of polycystic ovary syndrome (PCOS).

Efforts to establish phytomedicine traditions in modern drug discovery have been increasing in recent years. Few preclinical studies have investigated the effects of traditional plants in animal models. In one of these studies, Küpeli Akkol et al. demonstrated the beneficial effects of *A. mollis* and *A. persica* extracts in a rat endometriosis model [6]. According to their findings, the beneficial effects of these *Alchemilla* species could be partly attributed to their antioxidant and anti-inflammatory activities. Takır S et al. investigated the effects of *A. vulgaris* extract on vascular function in L-NAME-induced hypertensive rats, demonstrating that the extract of *A. vulgaris* caused vasorelaxant and blood pressure-lowering effects in these rats [7]. These effects may contribute to vasoprotection of vascular endothelium, which could be impaired in patients with PCOS.

Polycystic ovary syndrome is an endocrinopathy influenced by environmental, genetic, and hormonal factors. The pathophysiology of PCOS includes ovarian dysfunction, insulin resistance, hyperinsulinemia, hyperandrogenism, abnormal gonadotropin production, and hypothalamic-pituitary dysfunction [8]. Previous studies have shown that women with PCOS have an increased risk of low-grade chronic inflammation, insulin resistance, metabolic syndrome, type 2 diabetes, and dyslipidemia [9]. According to Christakou and Diamanti-Kandarakis, these factors increase the risks of hypertension, atherosclerosis, and endothelial dysfunction [10]. Insulin resistance and hyperandrogenism are key factors associated with vascular endothelial dysfunction and atherosclerosis in PCOS patients [11, 12]. Furthermore, measurement of urinary albumin excretion in women with PCOS is associated with cardiovascular risk factors and may provide clinically useful data for adverse cardiovascular events [13].

Human PCOS has been modelled in various rat models using hormonal methods, physiological manipulation, and genetical modification, to replicate many phenotypes of human disorder, including hyperandrogenism, LH elevation, polycystic ovaries, and insuline resistance [14].Hormonal methods using androgens and their derivatives are widely used to investigate alterations in endocrine biomarkers and ovarian morphology in PCOS. The rat model of PCOS induced by 5–6 weeks of treatment with dehydroepiandrosterone (DHEA) mimics several characteristics of human PCOS, such as hyperandrogenism, acyclicity, abnormal ovarian follicle maturation, and anovulation [15] Serum levels of androgens, estrogens, and luteinizing hormone (LH) have been demonstrated to increase in this animal model [16, 17]. Endocrine and metabolic changes were also observed in this model in previous studies [18, 19]. Furthermore, some studies have demonstrated vascular endothelial dysfunction in this animal model. Due to these advantages, DHEA-induced rat model of PCOS was used in the present study.

This study aimed to investigate the effects of *A. vulgaris* extract on DHEA-induced PCOS animals, based on its ethnobotanical use within the *Alchemilla* L. for the treatment of gynecological diseases, and previous research demonstrating the vasculoprotective effect of the plant in hypertensive rat models. To explore the mechanisms of AVE in the DHEA-induced PCOS model, vascular endothelial function was examined by assessing vascular function and endothelial nitric oxide synthase (eNOS) levels in thoracic aorta. Renal function was also evaluated in relation to vascular function. Additionally, systemic blood pressure (SBP), insulin, leptin, anti-Müllerian hormone (AMH), dihydrotestosterone (DHT), estrogen, glucose, and cholesterol levels in serum and ovarian tissue were analyzed to determine the effects of AVE on metabolic and reproductive disturbances.

## Materials Methods

### Plant Material and Preparation of the Extract

*Alchemilla vulgaris* L. plants at the flowering stage were collected from Zigana Mountain near Trabzon, Turkey, on 06.08.2023, (at an altitude of 1750–1900 m). The taxonomic identification of the plant materials was confirmed by H. Aşkın Akpulat, a senior plant taxonomist from the Department of Biology, Cumhuriyet University, Sivas, Turkey. To prevent harm to this species due to its unique nature, the collection was conducted with care, using limited materials. A voucher sample was deposited in the Herbarium of the Department of Biology, Cumhuriyet University, Sivas, Turkey (CUFH-Voucher no.: AA 8034). To prepare an infusion of the plant, 5 g of dried herb was added to 1000 mL of hot water and left for 10 min. After filtration, AVE extract was administered orally to the rats at a daily dose of 50 mg/100 g body weight [1].

### Animal Model

Thirty female Wistar albino rats (3 weeks old) were obtained from Kocaeli University, Experimental Medical Research and Application Center (Kocaeli, Turkey) and housed under standard laboratory conditions (room temperature 22 ± 2 °C, 12-h light/12-h dark cycle) with free access to foot pellets and tap water. This study was approved by the Kocaeli University Animal Research Ethics Committee (Project Number: KOU HADYEK 4/10–2016, Kocaeli, Turkey), in accordance with the Regulations of the Animal Research Ethics Committee in Turkey (number 26220, July 6, 2006).

The rats were randomly divided into three groups (*n* = 10 per group): control, PCOS, and PCOS + AVE groups. DHEA was dissolved in sesame oil and administered subcutaneously at a daily dose of 6 mg/100 mg/day for 35 days to the PCOS and PCOS + AVE groups, at a volume of 0.2 mL/100 g body weight.

Preliminary experiments were conducted to evaluate the effects of the vehicle solution on pharmacological and biochemical data. No statistical differences were found between sesame oil and saline treatments in the control animals. Therefore, animals in the control group received no treatment (data not shown).

*Alchemilla vulgaris* extract (AVE) was prepared daily and administered orally for 35 days to the PCOS + AVE group. The AVE dose was selected based on previous animal studies investigating the vascular and gyneacological effects of *Alchemilla* species [1, 6, 7]. *Alchemilla vulgaris* has a long history of use in traditional medicine for female reproductive health. Preclinical safety evaluations have reported that AVE is well-tolerated, with no known toxicity even at high doses [1, 20]. The effective therapeutic dose varies according to extraction method, route of administration, and pharmacological target; however, oral doses in the range of 50–500 mg/kg/day are commonly used in rodent models [1]. Accordingly, an oral dose of 500 mg/kg/day was selected in the present study to ensure both safety and sufficient biological activity. At the end of the 35-day PCOS period, body weight and systemic blood pressure of the rats were measured to confirm the induction of the experimental PCOS model as described in our previous study [18]. Blood and tissue samples were then collected under ketamine/xylazine (90 mg/kg/10 mg/kg) anesthesia for biochemical and pharmacological analysis.

### Blood Pressure Recording

Under ether anesthesia, polyethylene catheters (PE 10 attached to PE50) were inserted into the femoral artery with the other ends of the catheters (filled with heparinized saline) passed subcutaneously and externalized at the dorsal surface of the neck, where they were sutured to the skin. The Rats were allowed to recover from anesthesia for 2 h, after which the femoral artery catheter was used for continuous blood pressure monitoring for 60 min in freely moving rats. Systolic blood pressure (SBP) and diastolic arterial blood pressure (DBP) were recorded via a computer using the Biopac System MP 36 (St. Barbara, CA, USA). Mean arterial blood pressure (MABP) was calculated using the formula MABP = DBP + (SBP − DBP)/3.

### Organ Bath Studies

Following SBP measurement, the rats were sacrificed and thoracic aorta tissues were excised to assess vascular endothelial function, as previously described [18]. The thoracic aorta rings were prepared to approximately 5 mm in length and immediately placed in Krebs solution. The rings were mounted into 20-mL organ bath chambers containing Krebs solution, aerated with carbogen (95% O2 and 5% CO2), and maintained at 37 °C and pH 7.4. The isometric tension for each ring was measured using a force–displacement transducer (MAY-COM FDT 10 A,Commat Iletisim). A four-channel transducer data acquisition system was used to record the isometric forces of the rings (MP30B-CE; Biopac Systems, Santa Barbara, CA, USA) and analyzed with BSL Pro 3.7 software (Biopac Systems). The thoracic aorta preparations were allowed to equilibrate to a 1-g resting tension for 1 h, with the rings being washed with Krebs solution every 15 min to maintain the resting tension at 1 g throughout the experiment.

To check the viability of the preparations, thoracic aortas were contracted with 80 mM potassium chloride (KCl). The tissues were then washed and pre-contracted with a submaximal concentration of phenylephrine (3. 10^−6^ M—10^−5^ M). The relaxant responses to carbachol (10^–8^ −10^–5^ M), sodium nitroprusside (SNP; 10^–9^ −10^–4^ M), or papaverine (10^–4^ M) were obtained from precontracted aortic rings. After completing each concentration–response curve, the tissues were rinsed with fresh buffer and allowed to return to basal tension for 30 min.

### Measurement of Serum Hormones and TNFα

At the end of the 35-day DHEA and/or AVE administration period, blood samples were collected and immediately centrifuged at 4000 rpm for 15 min. The separated serum samples were stored at −80 °C until futher analysis. Serum concentrations of TNF-α, glucose, insulin, leptin, cholesterol, dihydrotestosterone (DHT), estradiol, and anti-Müllerian hormone (AMH) were measured to evaluate the effects of PCOS using enzyme-linked immunosorbent assays (Sunbred, Baashan district, Shangai), according to the manufacturer’s instructions. The samples were analyzed using a microelisa reader (Alisei Quality System Seac, RADIN Company).

### Measurement of Urinary Protein and Albumin Excretion

Urine samples from the rats (*n* = 5/group) were collected in plastic metabolic cages over a 24 h. Urinary albumin and protein excretion levels were measured using an ELISA reader (Abbott Architecht c16000).

### Ovarian and Vascular Immunohistochemistry

#### Hematoxylin & Eosin (H&E) Staining

Evaluation of ovarian architecture and follicle count was performed using hematoxylin–eosin (H&E)-stained sections. H&E staining was applied to every tenth section of the ovary, with each section separated by approximately 50–60 μm from the next section. All follicle types were classified and counted as previously described [18]. All the sections were evaluated using an upright bright-field optical microscope (Leica DM 1000) and documented using an attached digital camera (Leica DMC 2900).

#### eNOS and TNF-α Immunohistochemistry

Aortic and ovarian tissue samples were deparaffinized using toluene and rehydrated with a descending series of ethanol solutions. After heat-induced antigen retrieval, sections were rinsed with Tris-buffered saline (TBS). Endogenous peroxidase activity was inhibited by treating the samples with 3% hydrogen peroxide solution. The samples were then washed twice with TBS and blocked with protein-blocking solution. Subsequently, the sections were incubated overnight with rabbit polyclonal antibody against TNF-α (ab183896, Abcam; 1:750 dilution). Aortic sections were labeled with a mouse monoclonal anti-eNOS primary antibody (ab76198, Abcam; 1:1000 dilution) overnight. After incubation with primary antibodies, the specimens were immersed in TBS-Tween 20 (TBS-T) and incubated with HRP-conjugated secondary antibodies for 15 min at room temperature. After washing with PBS, the sections were incubated with DAB and counterstained with Mayer's hematoxylin. The samples were analyzed under an optical microscope (Leica DM 1000) equipped with a digital camera (Leica DMC 2900). The expression intensity of TNF-α and eNOS was quantified using ImageJ software.

### Drugs and Solutions

DHEA, phenylephrine hydrochloride, carbachol, SNP, and papaverine hydrochloride were obtained from Sigma-Aldrich Co. (St. Louis, MO, USA). DHEA was dissolved in sesame oil. All drugs, except DHEA, were freshly prepared in distilled water, and kept on ice during the experiments. For organ bath studies, Krebs Solution was used with the following ionic composition (mM): NaCl 118, KCl 4.71, MgCl_2_ 1.05, NaH_2_PO_4_ 1.33, NaHCO_3_ 25, CaCl_2_ 2.7, and glucose 5.6. In the high-K^+^ solution, NaCl was replaced with equimolar amounts of KCl. An aqueous extract of *A. vulgaris* was used at a concentration of 5 mg/mL.

### Statistical Analysis

All data are presented as mean ± standard error of the mean (SEM). Mean arterial blood pressure (MABP) was evaluated using the Friedman test, followed by post-test Dunn's Multiple Comparison Test and one-way analysis of variance (ANOVA). Differences in repeated measurements within the group and between groups at the 5 th, 15 th, 30 th, and 60 th minutes were analyzed using Bonferroni's Multiple Comparison Test. In organ bath studies, the relaxant responses to carbachol and SNP were expressed as a percentage of the precontraction induced by phenylephrine. Differences between experimental groups were determined using one-way ANOVA followed by Tukey’s post hoc-test. Immunoreactivity scores were evaluated using the Kruskal–Wallis test, followed by Dunn’s multiple comparison test. Statistical significance was set at *p* < 0.05.

## Results

### Induction of Rat PCOS Model and Improved Phenotypes

**Fig. 1:**
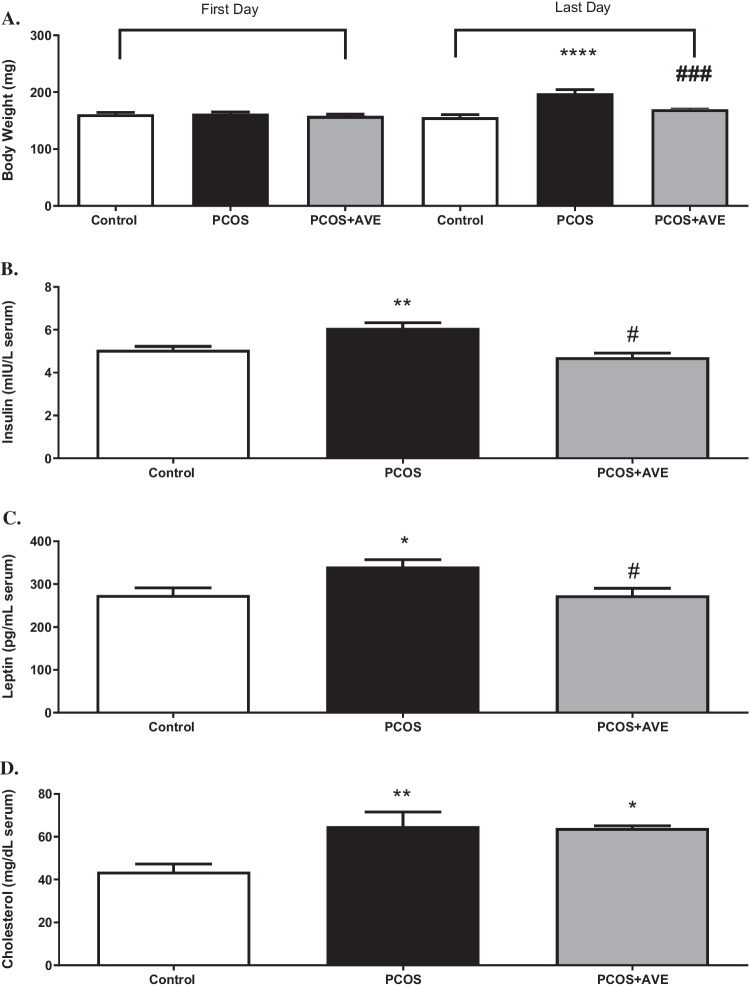
Metabolic changes in the control, PCOS, and PCOS + AVE groups. **A** Body weight, **B** Serum insulin levels, **C** Serum leptin levels, **D**: Serum cholesterol levels. Values are expressed as mean ± S.E.M. * *p* < 0.05, **p < 0.01, **** *p* < 0.0001 compared with the control group; #*p* < 0.05, ###*p* < 0.001 compared with the PCOS + AVE group

**Fig. 2:**
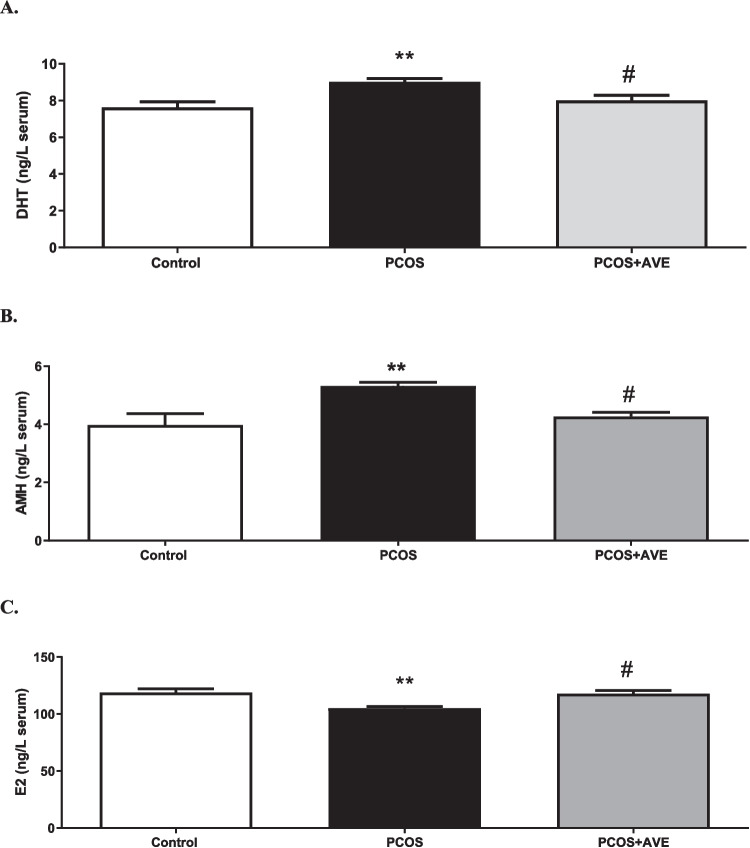
Sex hormon levels in the control, PCOS, and PCOS + AVE groups. **A** Serum DHT, **B** Serum AMH, **C**-Serum E2. Values are expressed as mean ± S.E.M. **p < 0.01, compared with the control group; #*p* < 0.05, compared with the PCOS + AVE group

PCOS induction was confirmed by measuring body weights and hormonal changes in the DHEA-induced PCOS rat model. The average body weight of the PCOS group was significantly higher than that of the control group (Fig. 1A) (*p* < 0.05). Serum levels of DHT and AMH were increased, while E2 levels were decreased in the PCOS group compared to the controls (Fig. 2A, B, C) (*p* < 0.05). Serum insulin, leptin, and cholesterol levels were measured to assess the metabolic phenotypes of the model. DHEA administration induced significant increases in metabolic parameters, reflecting the metabolic dysfunction observed in human PCOS (Fig. 1B, C, D) (*p* < 0.05). The observed hormonal changes and elevated body weight in the PCOS group indicated the successful establishment of the PCOS model with following a 35-day DHEA injection. In addition to these metabolic and endocrine changes, polycystic ovarian morphology was verified by histological examination. H&E-stained ovarian sections revealed multiple atretic follicles, reduced numbers of Graafian follicles and corporus luteum, and impaired folliculogenesis. These histological findings are consistent with previously described polycystic-like ovarian changes in DHEA-induced PCOS models.

AVE treatment reversed the average body weight and serum levels of DHT, AMH, and E2 in the PCOS + AVE group to levels similar to those observed in the control group (Figs. 1A and 2A, B and C). In addition, the PCOS + AVE group exhibited significantly decreased serum levels of insulin, leptin, and cholesterol (*p* < 0.05) compared to the PCOS animals (Fig. 1B, C, D).

### Urinary Protein and Albumin Excretion

**Fig. 3:**
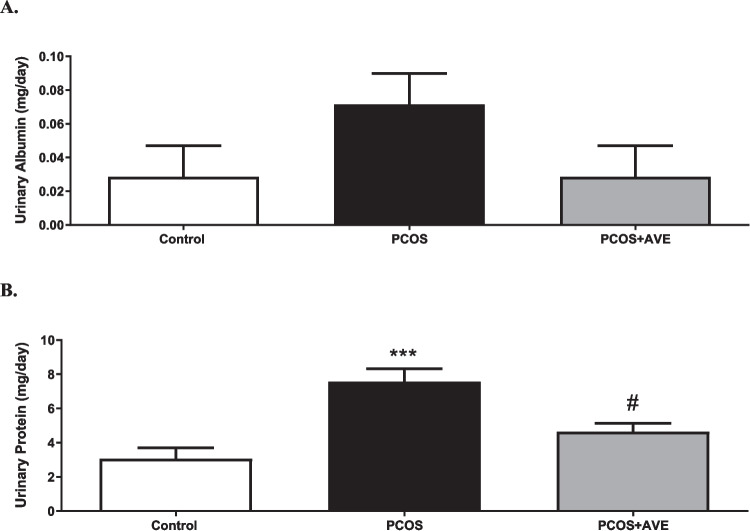
Renal function in the control, PCOS, and PCOS + AVE groups. **A**: Urinary albumin levels, **B**: Urinary protein levels. Values are expressed as mean ± S.E.M. ***p < 0.001 compared with the control group; #*p* < 0.05 compared with PCOS + AVE group

The effects of PCOS and AVE treatment on renal function were assessed by measuring urinary albumin and protein excretion. Urine albumin levels were similar in all groups (Fig. 3A). However, urinary protein levels were elevated in the PCOS group and returned to reversed to control levels after AVE treatment (*p* < 0.05) (Fig. 3B).

### SBP and Vascular Reactivity

**Fig. 4:**
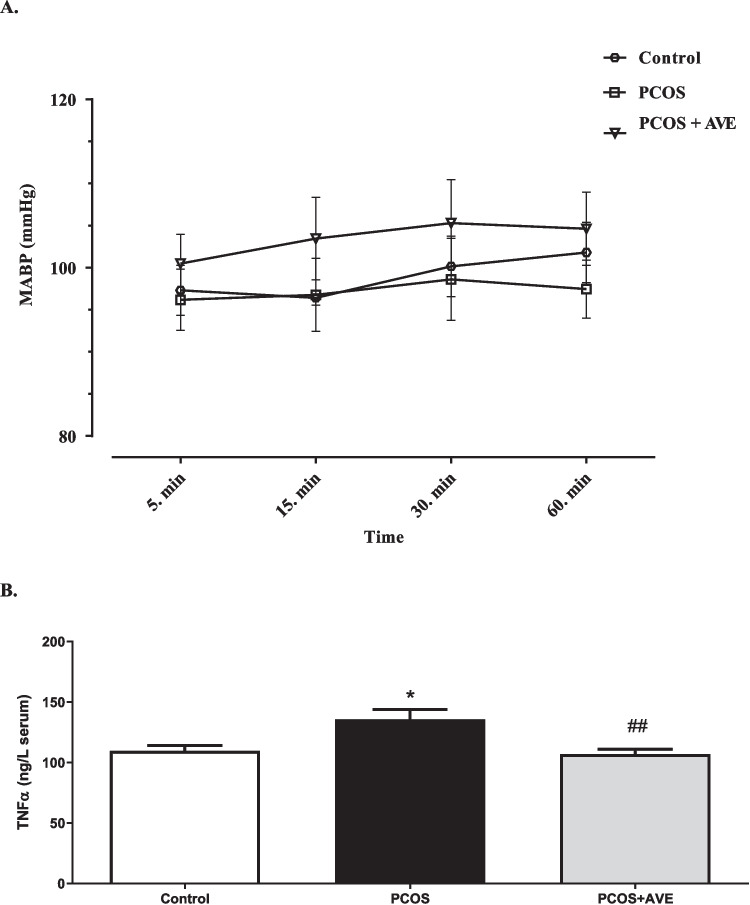
SBP (mean arterial blood pressure (MABP, mmHg) (**A**) and Vascular TNF-α levels (**B**) in the Control, PCOS, and PCOS + AVE groups

**Fig. 5:**
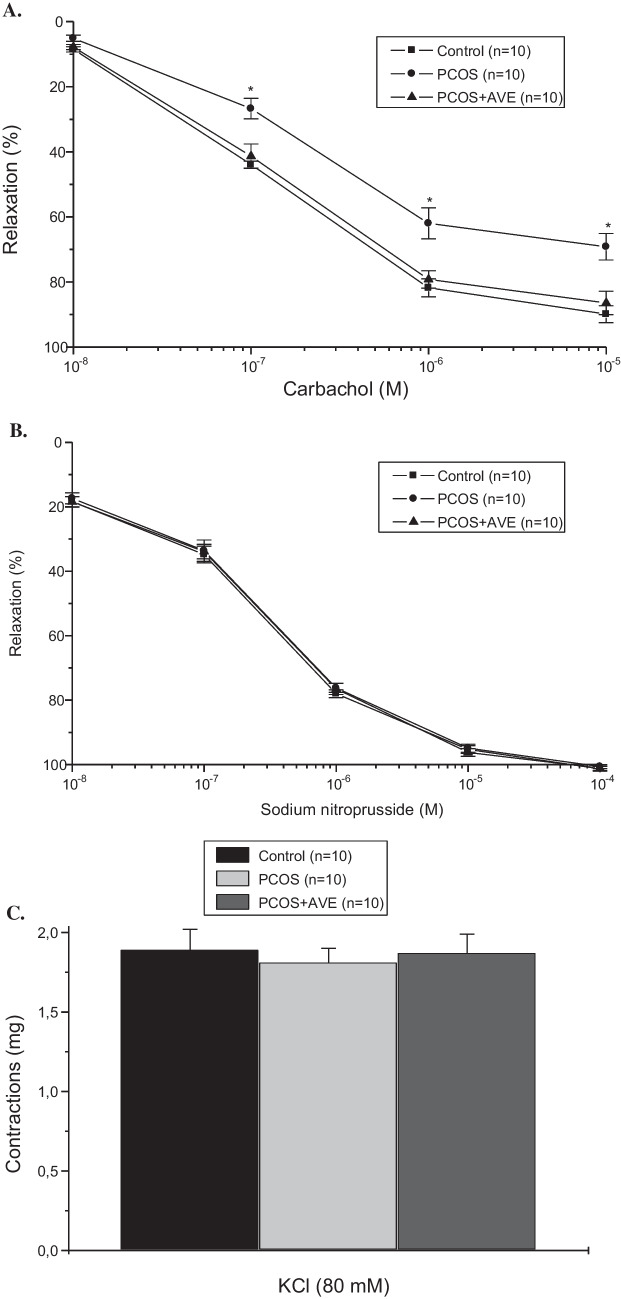
Carbachol-induced endothelium-dependent relaxation responses (**A**) SNP-induced-endothelium-independent relaxation responses (**B**), and KCl (80 mM)- induced contractile responses (**C**) in the control, PCOS, and PCOS + AVE groups. Values are expressed as mean ± S.E.M.; *n* = number of animals used. *p < 0.05 compared with control group; #*p* < 0.05 compared with the PCOS + AVE group

There was no significant difference in repeated SBP measurements between the groups (Fig. 4A). Differences in repeated measurements within group and between groups at the 5 th, 15 th, 30 th, and 60 th minutes were also similar. The contractile responses to 80 mM KCl (Fig. 5C) and precontraction with phenylephrine (10^–5^−3.10^−6^ M) of the rings were comparable across all groups (data not shown). Relaxant responses were evaluated in precontracted thoracic aorta rings. Endothelium-dependent relaxation in response to carbachol was significantly decreased in the PCOS group (*p* < 0.05). However, this impairment was reversed to control levels after AVE treatment (Fig. 5A). Endothelium-independent relaxant responses to SNP were similar across all groups (Fig. 5B). Additionally, there was no significant differences in the relaxant responses to papaverine between groups (data not shown).

### TNF-α -an Inflammatory Biomarkers

Serum TNF-α levels, an indicator of inflammation, were significantly increased in the PCOS group compared to the control group (*p* < 0.05) (Fig. 4B). Following AVE treatment, these elevated TNF-α levels were normalized to values comparable to the control group.

### Ovarian and Vascular Immunohistochemistry

#### H&E Staining

**Fig. 6:**
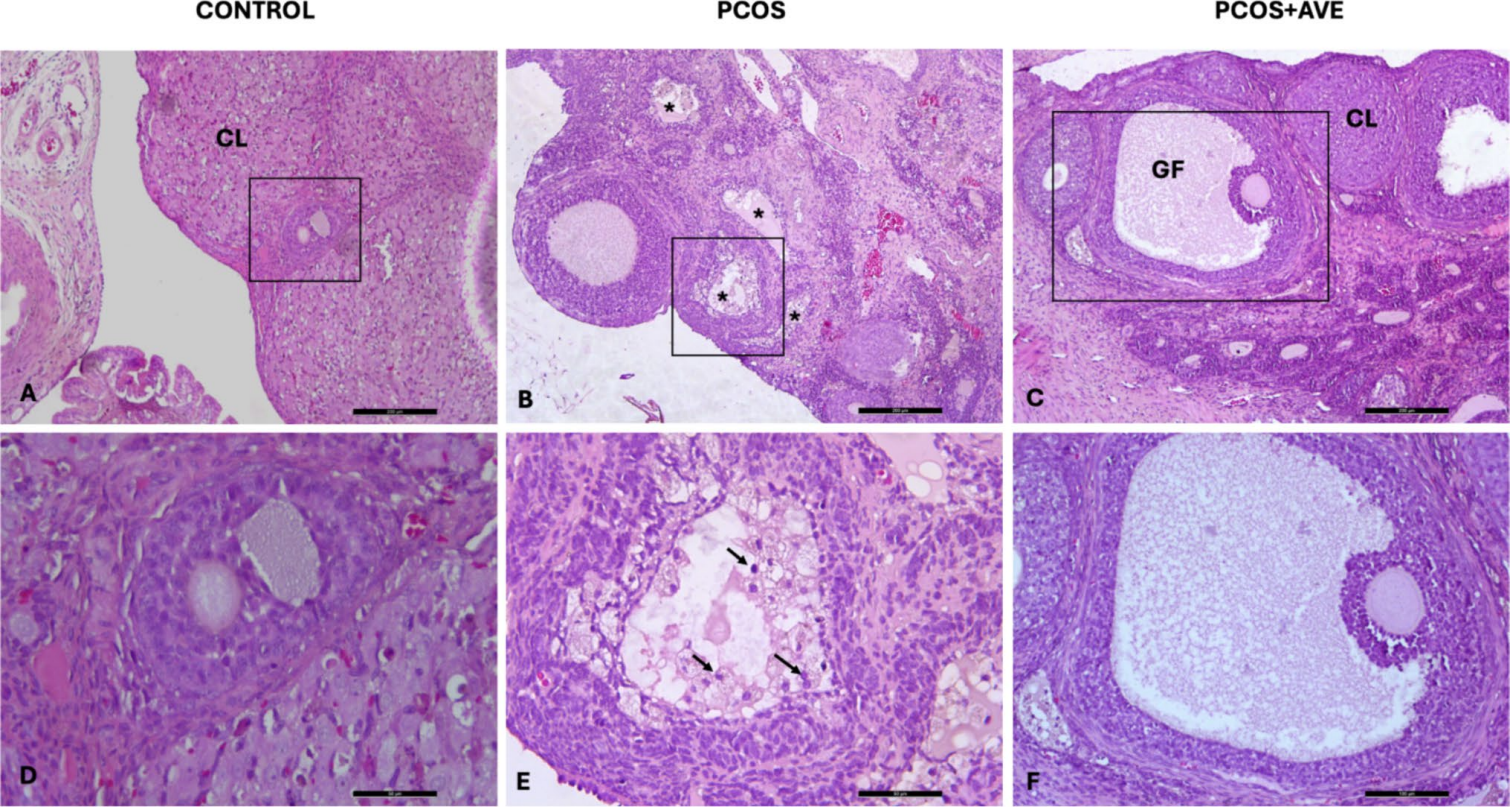
Histopathological alterations of ovary in the control (**A**, **D**), PCOS (**B**, **E**), and PCOS + AVE (**C**, **F**) groups. Many atretic follicles (Fig. 6B-asterix) were observed in the PCOS group. Arrows indicate granulosa cells with pycnotic nuclei in atretic follicles. CL: Corpus luteum; GF: Tertiary follicle. (A-C, 40X magnification; D-E, 100X magnification; F, 200X magnification, H&E)

Ovarian sections from the control group displayed normal ovarian morphology, exhibiting all types of follicles at different stages of folliculogenesis along with the presence of the corpus luteum, which is a sign of ovulation. The oocytes and their surrounding zona pellucida showed no degeneration (Fig. 6A and D). However, the PCOS group exhibited many atretic follicles (Fig. 6B, indicated by stars). Granulosa cells with pycnotic nuclei were observed in the antrum of these atretic follicles (Fig. 1E, indicated by arrows). In the AVE treatment group, ovarian morphology improved to resemble that of the control group, as indicated by an increased number of corpus luteum and Graafian follicles, and decreased number of atretic follicles (Fig. 6C and F). Additionally, oocyte and the surrounding zona pellucida of Graafian follicles were normal and intact (Fig. 6F).

In H&E-stained sections, follicle counts were analyzed to determine the follicle pool. Compared to the control group, the number of primordial, unilaminar primary, multilaminar primary, Graafian follicles and corpus luteum decreased in the PCOS group but increased following AVE treatment. While the number of secondary follicles remained unchanged across all groups, the number of atretic follicles increased in the PCOS group compared to the control group and decreased in the PCOS + AVE group compared to the PCOS group. No significant differences were observed between the control and PCOS + AVE groups in terms of secondary follicles, atretic follicles, and corpus luteum. The decrease in atretic follicles, along with the increased number of Graafian follicles and corpus luteum in the PCOS + AVE group, suggests that AVE treatment ameliorated PCOS-induced impairment in folliculogenesis (Table 1).

**Table 1:**
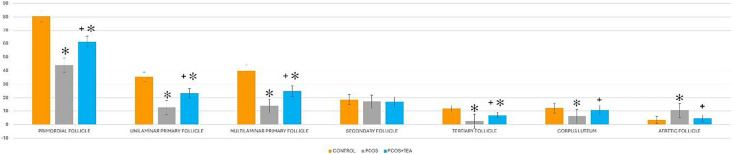
Mean ± SEM number of ovarian follicles in Control, PCOS, and PCOS + AVE groups

^*^indicate significance from the Control group at *p* < 0,05 probability level

+ indicate significance from the PCOS group at *p* < 0,05 probability level

**Fig. 7:**
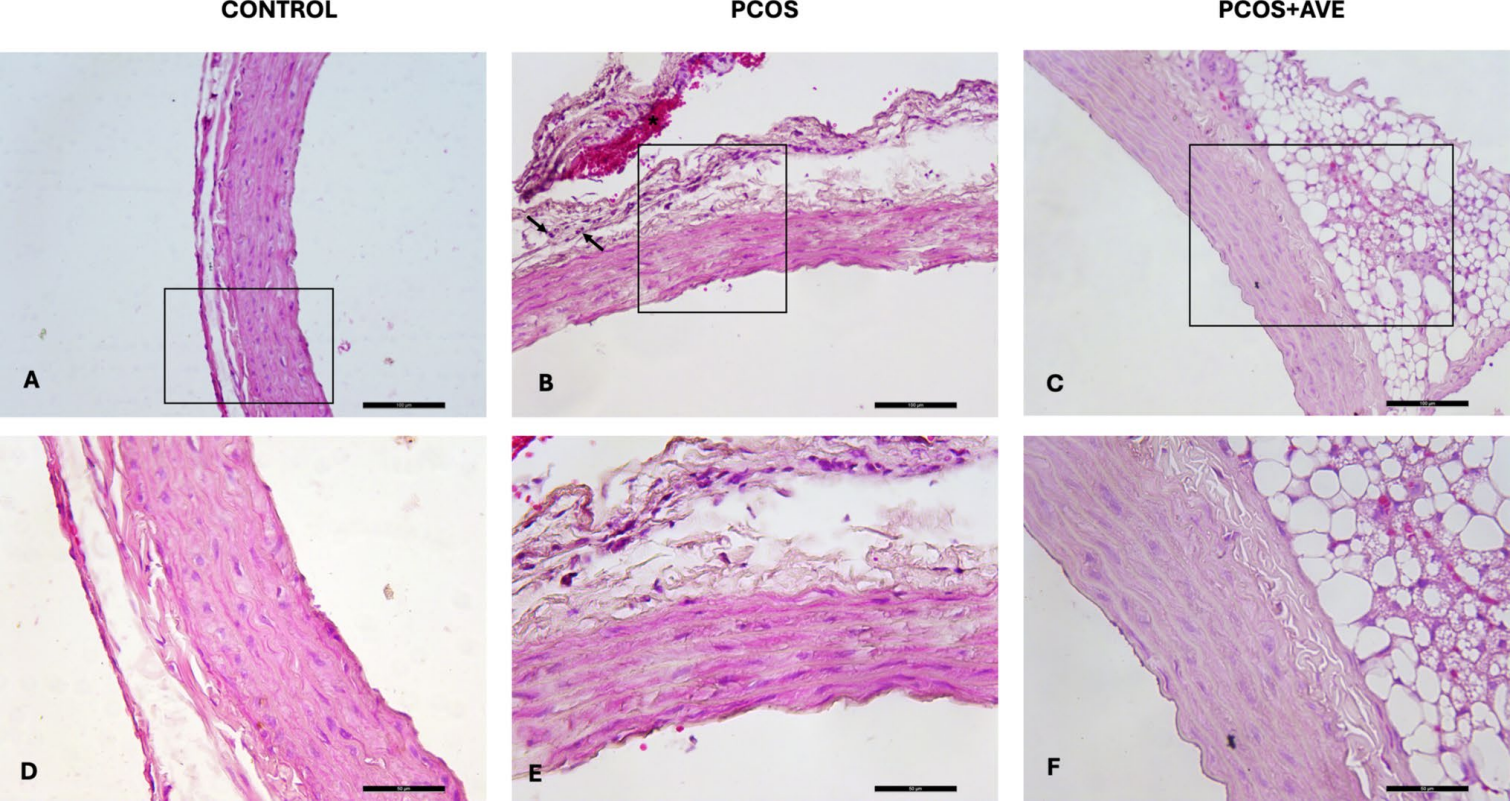
Histopathological alterations of the thoracic aorta in the control (**A**, **D**), PCOS (**B**, **E**), and PCOS + AVE (**C**, **F**) groups. Note the leukocytic infiltration (arrows) and hemorrhage (black star) in the PCOS group. (A-C, 200X magnification; D-F, 400X magnification, H&E)

Thoracic aorta sections from the control group exhibited healthy morphology and normal thickness of the intima, media, and adventitia layers (Fig. 7A and D). In contrast, the PCOS group displayed an irregular media, detachment between the media and adventitia, leukocytic infiltration (Fig. 7B, indicated by arrows), and hemorrhage in the adventitia (Fig. 2B, indicated by star) compared to the control group. In the PCOS + AVE treatment group, aortic morphology and architecture was restored to comparable levels to the control group (Fig. 7C and F).

### TNF- α and eNOS Immunoreactivity

**Fig. 8:**
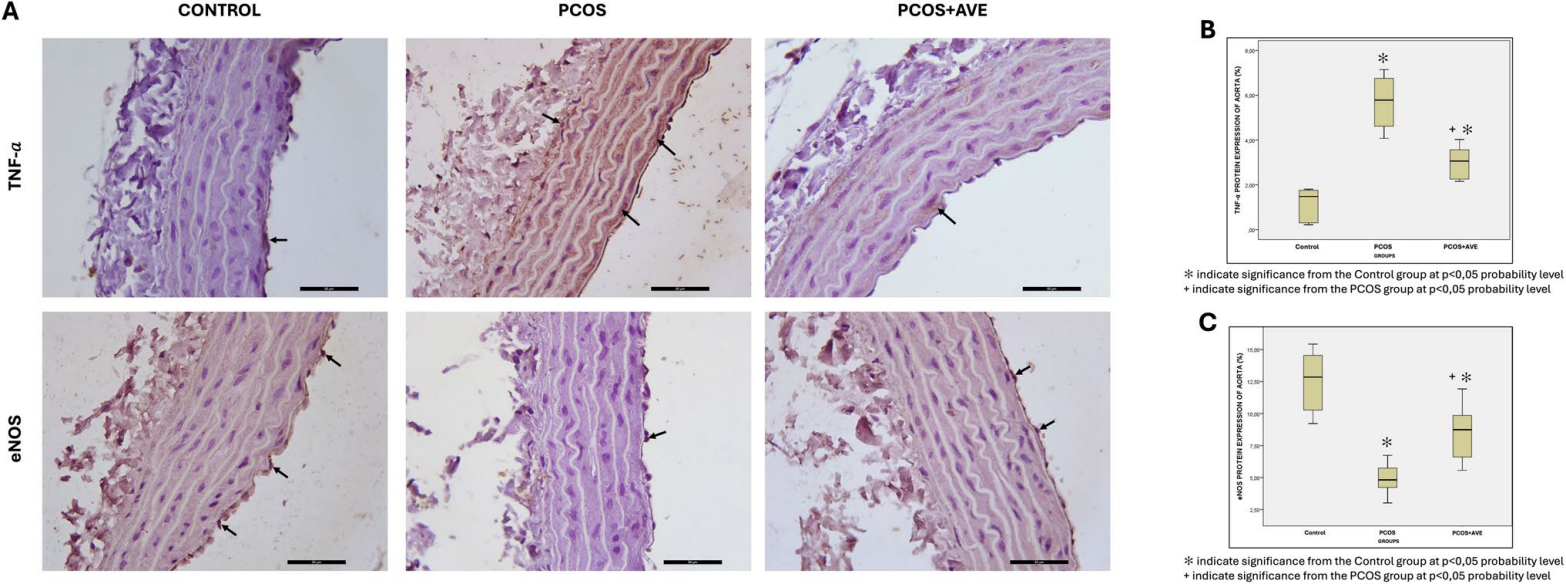
**A** Immunohistochemical staining of aortic eNOS and TNF-α in the control, PCOS, and PCOS + AVE groups. **B** TNF-α and (**C**) eNOS expression levels in all groups. Black arrows indicate eNOS or TNF-α-positive cells. 400 × magnification

**Fig. 9:**
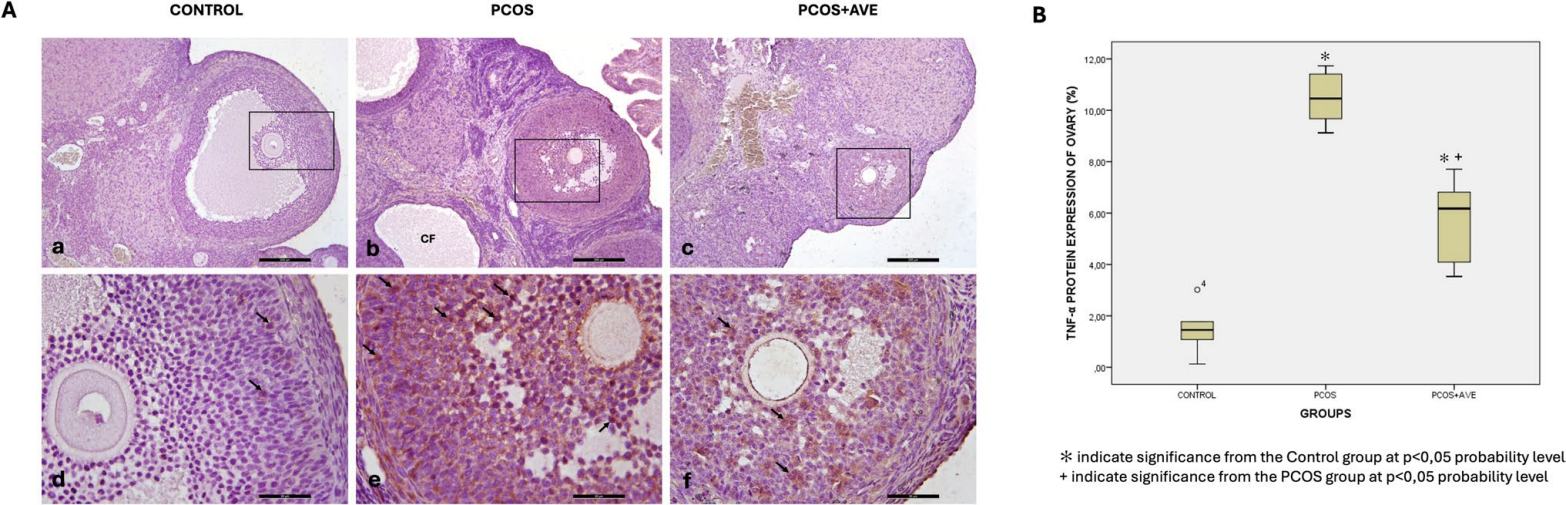
**A** Immunohistochemical staining of ovarian TNF-α in the control (**a**-**d**), PCOS (**b**-**e**), and PCOS + AVE (**c**-**f**) groups. **B** TNF-α expression levels in all groups. Black arrows indicate TNF-α-positive cells. a-c:100X; d-f: 400 × magnification

In the aorta and ovary, immunohistochemical staining intensities of TNF-α and eNOS were quantified using ImageJ software. In aortic tissue sections, the expression of TNF-α and eNOS in the PCOS and PCOS + AVE groups showed statistically significant differences compared to the control group. In the PCOS + AVE group, TNF-α expression levels decreased, while eNOS expression levels increased compared to those in the PCOS group (Fig. 8). In ovarian sections, TNF-α expression levels were higher in both the PCOS and PCOS + AVE groups compared to the control group. However, AVE treatment significantly reduced TNF-α expression compared to the PCOS group (Fig. 9).

## Discussion

In the present study, DHEA-induced PCOS animals exhibited vascular endothelial dysfunction, hormonal imbalances, and ovarian morphological changes, all of which improved after AVE treatment. Moreover, AVE reduced ovarian and aortic TNF-α levels, which were elevated in the PCOS group. Our findings suggest that the anti-inflammatory and vasculoprotective effects of AVE may be beneficial in the treatment of PCOS.

Several animal models have been developed to stimulate the hormonal and ovarian changes in patients with PCOS [14]. In this study, a rat model of DHEA-induced PCOS was used to replicate ovarian and metabolic characteristics similar to those observed in women with PCOS. The model was confirmed by measuring metabolic and endocrine biomarkers in the blood. Elevated levels of DHT and AMH, along with decreased levels of E2, were observed as hormonal changes, while increased levels of insulin, leptin, and cholesterol were noted as metabolic changes in the PCOS group. An increase in average body weight was also observed in the PCOS group. These changes reflect the metabolic and endocrine phenotypes of PCOS in humans. In addition, the histological findings, such as a decrease in Graffian and corpus luteum follicles and an increase in cystic, secondary, and atretic follicles further validated the model.

*Alchemilla*, a genus of herbs in the Rosaceae family, has been traditionally used as a folk medicine in some countries. *A. vulgaris*, a well-known species in *Alchemilla* genus, has been widely used for treatment of gynecological conditions such as dysmenorrhea, menorrhagia, and menopausal complaints, traditionally [1, 3, 4]. It has also been used in folk medicine for female infertility [5]. Despite its widespread traditional use, there are limited studies investigating the mechanisms of action of the *Alchemilla* genus in experimental models of gynecological diseases. Küpeli Akkol et. al. Demonstrated that the extract of *A. Mollis* and *A. Persica* have beneficial effects in an animal model of endometriosis, reporting a reduction in the severity of endometriotic lesions after treatment [6]. Additionally, they found that TNF-α and IL-6 levels were reduced in *Alchemilla*-treated endometriosis group. A relationship between elevated levels of pro-inflammatory cytokines and ovarian dysfunction has been demonstrated in a PCOS rat model [21, 22]. Elevated TNF-α levels plays a key role in the development of PCOS. In our previous study, we showed that treatment with etanercept, an anti-TNF-α drug, exhibited beneficial effects in PCOS. The beneficial effect of etanercept was partly attributed to decreased ovarian TNF-α expression in treared rats [18]. Similarly, in the present study, the AVE may have exerted beneficial effects by reversing the inflammation seen in PCOS. Furthermore, the improvement in ovarian morphology following AVE treatment may be due to its anti-inflammatory properties.

Previous studies have documented a relationship between PCOS and vascular endothelial dysfunction [10, 23]. It has been shown that women with PCOS exhibit endothelial inflammation, endothelial cell proliferation, and coagulation disorders, all of which contribute to endothelial dysfunction and subsequently to atherosclerosis [10]. Furthermore, patients with PCOS often present with hormonal abnormalities, including hyperandrogenism and elevated insulin levels, which can independently contribute to vascular endothelial dysfunction and exacerbate the risk of cardiovascular disease [24, 25]. Several studies have found that correlation between hyperandrogenemia, insulin resistance and endothelial dysfunction in PCOS patient [24, 26]. Chronic inflammation has also been reported as another contributing factor to vascular endothelial dysfunction [18, 27] Consistent with our and previous studies, alongside metabolic impairments and vascular endothelial dysfunction, increased level of TNF-α in serum and vascular tissue was observed in the PCOS group. The PCOS-related metabolic and vascular changes was improved after AVE treatment. AVE’s anti-inflammatory and vasorelaxant effects may have synergistically prevented the changes caused by PCOS.

Extracts from various plant species in the *Alchemilla* genus have been traditionally used for the treatment of hypertension. The cardiovascular effects of a limited number of species within the *Alchemilla* genus have also been investigated in some in vitro studies. In a previous study, it was shown that an extract of *A. viridiflora* exhibited an in vitro inhibitor effect on angiotensin-converting enzyme, a major contributor for hypertension [28]. In another in vitro study, the effects of *A. vulgaris* extract on vascular function were investigated in L-NAME-induced hypertensive rats. Takir et. al. demonstrated that the extract of *A. vulgaris* caused vasorelaxant and blood pressure-lowering effects in these rats [7]. They concluded that these beneficial effects of *A. vulgaris* may contribute to vasoprotection of vascular endothelium, which is often impaired in patients with PCOS. Consistent with these studies, treatment with AVE was shown to reverse PCOS-induced vascular endothelial dysfunction in the present study. Thus, AVE may have a protective effect against PCOS-induced endothelial dysfunction. The vasculopretective properties of AVE may contribute to its therapeutic potential for the treatment of women with PCOS.

Several studies have reported that microalbuminuria is more common in women with PCOS than in healthy women. Furthermore, clinical studies have suggested a possible link between cardiovascular risk factors and elevated albumin excretion in the urine in patients with PCOS [13, 29]. In present study, urine albumin and protein levels were evaluated to assess renal function in the PCOS group, and no statistically significant differences were found in urinary albumin and protein levels. Although consistent findings were observed across all groups under our experimental conditions, further studies are needed to investigate the role of microalbuminuria in DHEA-induced PCOS animal model.

## Conclusion

The present study demonstrated that the DHEA-induced PCOS animal model exhibited endocrine, metabolic, and vascular changes, with increased inflammation. These impairments may lead to changes in ovarian morphology. AVE treatment reversed the PCOS-induced hormonal disturbances, vascular endothelial dyfunction, and ovarian morphological changes. AVE may have therapeutic potential due to its anti-inflammatory and vasorelaxant effects in patients with PCOS.

## Data Availability

The data underlying this article will be shared on reasonable request to the corresponding author.
